# Upstream ORFs Influence Translation Efficiency in the Parasite *Trypanosoma cruzi*

**DOI:** 10.3389/fgene.2020.00166

**Published:** 2020-02-28

**Authors:** Santiago Radío, Beatriz Garat, José Sotelo-Silveira, Pablo Smircich

**Affiliations:** ^1^Department of Genomics, Instituto de Investigaciones Biológicas Clemente Estable, Ministerio de Educación y Cultura, Montevideo, Uruguay; ^2^Laboratory of Molecular Interactions, Facultad de Ciencias, Universidad de la República, Montevideo, Uruguay; ^3^Department of Cell and Molecular Biology, Facultad de Ciencias, Universidad de la República, Montevideo, Uruguay

**Keywords:** *Trypanosoma cruzi*, uORF, 5′UTR, translation efficiency, translation regulation

## Abstract

It is generally accepted that the presence of ORFs in the 5′ untranslated region of eukaryotic transcripts modulates the production of proteins by controlling the translation initiation rate of the main CDS. In trypanosomatid parasites, which almost exclusively depend on post-transcriptional mechanisms to regulate gene expression, translation has been identified as a key step. However, the mechanisms of control of translation are not fully understood. In the present work, we have annotated the 5′UTRs of the *Trypanosoma cruzi* genome both in epimastigotes and metacyclic trypomastigotes and, using a stringent classification approach, we identified putative regulatory uORFs in about 9% of the analyzed 5′UTRs. The translation efficiency (TE) and translational levels of transcripts containing putative repressive uORFs were found to be significantly reduced. These findings are supported by the fact that proteomic methods only identify a low number of proteins coded by transcripts containing repressive uORF. We additionally show that AUG is the main translation initiator codon of repressive uORFs in *T. cruzi*. Interestingly, the decrease in TE is more pronounced when the uORFs overlaps the main CDS. In conclusion, we show that the presence of the uORF and features such as initiation codon and/or location of the uORFs may be acting to fine tune translation levels in these parasites.

## Introduction

Translation regulation depends on signals that are present mainly at the untranslated regions of the mRNAs (UTRs). Although many regulatory elements are present in the 3′UTR regions, 5′UTRs may also contain important *cis* acting regulators. In particular, the presence of small open reading frames (upstream ORFs, uORF) in this region has been described as a regulatory mechanism influencing the formation of the translation initiation complex at the initiator codon of the main CDS (McCarthy, 1998). Typically, the presence of uORFs decreases the efficiency of initiation at the main CDS, thus leading to downregulation of its translation rate (Griffin et al., 2001; Vattem and Wek, 2004; Chen et al., 2010). Even though there are cases where the presence of a uORF positively influences the translation efficiency (TE) of the main CDS, these are exceptions (Griffin et al., 2001; Vattem and Wek, 2004; Chen et al., 2010). uORFs may serve as elements that respond to altered environmental conditions, allowing the cells to rapidly adjust their protein production rates (Calvo et al., 2009; Lawless et al., 2009). Features of the uORFs that are efficiently translated and negatively regulate the main CDS have been defined. Among them, the uORF position within the 5′UTR, its length, the sequence context of the initiation codon and the overlap with the main CDS have all been shown to affect their regulatory potential (Kozak, 1987, 2002; Rajkowitsch et al., 2004; Wethmar, 2014; Chew et al., 2016; Fervers et al., 2018).

Kinetoplastids are excellent models for the analysis of post-transcriptional mechanisms of gene expression regulation, given that transcription initiation is considered to be constitutive for most of the genome (Soldatos et al., 2010). This implies that the control of the substantial and rapid cell biology changes that take place throughout their life cycle, must rely mostly on post-transcriptional mechanisms (Soldatos et al., 2010). In addition to studies on regulation of mRNA steady state levels and protein degradation or modification, ribosome profiling approaches (Ingolia et al., 2011; Ivanov et al., 2011; Smircich et al., 2015) have confirmed that translation is a key step to regulate gene expression profiles in these organisms (Peabody, 1989; Vasquez et al., 2014; Smircich et al., 2015).

The role of uORFs in kinetoplastids has been studied using reporter genes and through the analysis of Ribo-Seq and proteomic data (Siegel et al., 2005; Jensen et al., 2014; Vasquez et al., 2014; Fervers et al., 2018). Probably due to the use of different definitions, the number of CDS associated to uORFs in the kinetoplastid genomes remains controversial, ranging from 11–22% in *T. brucei* (according to (Jensen et al., 2014) or Siegel et al., 2005, respectively) or 29% in the *T. congolense* (according to Fervers et al., 2018).

Except for one early report about uORFs composition in four specific genes (Jaeger and Brandao, 2011), a systematic analysis of uORFs in *Trypanosoma cruzi* is missing. This parasitic protozoan, is the causative agent of the neglected Chagas’ disease a neglected infection affecting millions of people in Latin America (Lidani et al., 2019). It has a complex life cycle involving replicative and non-replicative developmental forms both in vertebrate (amastigote and bloodstream trypomastigotes, respectively) and invertebrate hosts (epimastigotes and metacyclic trypomastigotes, respectively) (Tyler and Engman, 2001).

In the present study, we performed a systematic search for uORFs in *T. cruzi* 5′UTRs focusing on those with repressive potential. The two life cycle forms of the parasite in the invertebrate host (epimastigotes and metacyclic trypomastigotes) were analyzed to detect putative stage-specific control. Using a stringent approach that considers factors that influence the uORF TE, we found that at least 5% of the 5′UTRs of the mRNAs in the *T. cruzi* genome contain repressive uORFs (approx. 9% of the analyzed 5′UTRs). Our results show that genes containing these uORFs have a low translational efficiency and low translation levels in both epimastigotes and metacyclic trypomastigotes insect stages. We also show that the AUG codon is mainly responsible for initiating translation of the uORFs that cause this effect. Additionally, we analyzed the uORFs that overlap the main CDS, a category not previously analyzed in *T. cruzi*. We found that these overlapping uORFs (uORFo) are associated to the most pronounced decrease of translation efficiency in this organism. In conclusion, we present a repertoire of genes in *T. cruzi* that exhibit putative repressive uORFs at the 5′UTR and define characteristics such as the identity of the initiation codon and/or location of the uORFs, that may be contributing to the regulation of gene expression by fine tuning the translation levels.

## Materials and Methods

### Genomic Data

The genome sequence of *T. cruzi* strain CL Brener and its gene annotation were obtained from the TriTrypDB database (version 32).

### UTRs Determination

The UTR sequences were determined for the epimastigote and metacyclic trypomastigote stages using the UTRme software (Radio et al., 2018), based on the transcriptomic data (accession numbers ranging from SRR1346053–SRR1346059) data generated in Li et al. (2016). The 5′UTRs with the highest scoring *trans*-spliced site and longer than 5 nucleotides were used. 8206 5′UTR regions were annotated for epimastigotes and 8217 for metacyclic trypomastigotes.

Multigene family members were not analyzed because, due to either the large content of repeated regions or to assembly problems, the 5′UTR ends are difficult to assess. Besides, their high UTR similarity and abundance would bias the results. Multigene families removed include large families of surface proteins such as MASPs, GP63, Mucins, and TcTS. Finally, we also decided to remove 5′UTR that contain fragments of *T. cruzi* protein coding regions, likely produced by assembly or annotation errors. For this purpose, the BLASTX tool (Altschul et al., 1990) was used, eliminating UTRs that returned a hit against the *T. cruzi* CDS with an *e*-value of less than 0.005.

### uORF Annotation and Classification

Annotation of 5′UTR sequences was performed using the UTRme tool (Radio et al., 2018) using data obtained from Li et al. (2016). Open reading frames (ORF) greater than three codons were obtained using the getORF tool (Rice et al., 2000). For all ORFs present in the same strand as the CDS, AUGs in frame to the stop codon of the ORF (uAUG) were searched to define all putative coding sequences. uORFs in which the coding sequences start in the 5′UTR region and end within the main CDS (out of frame with respect to the main CDS AUG) were defined as overlapping uORFs (uORFo) while the ones contained entirely in the 5′UTR were defined as non-overlapping uORF (uORFno), in this case the frame with respect to the main CDS AUG was not considered.

Putative repressive uORFno elements were filtered considering the following characteristics: (1) the presence of an AUG start codon; (2) a minimum distance of 15 nucleotides from the uAUG to the 5′ end; (3) a maximum distance of 50 nucleotides between the stop codon of the uORF and the AUG of the main CDS; (4) minimum length of 5 amino acids. In the case of the uORFo the requirements were: (1) to start with AUG; (2) a minimum length of three amino acids before the AUG of the main CDS.

To identify and study uORFs with coding sequences initiating from near cognate codons (NCC), the annotation and classification strategy remained the same with the exception that the initiator codon was changed from AUG to the codon under study.

### Translation Efficiency Determination

Ribosome Profiling data were obtained from the SRA: PRJNA260933 (Smircich et al., 2015) and correspond to the epimastigote and trypomastigote metacyclic stages of *T. cruzi*. The cutadapt program (Martin, 2011) was used to remove the adapters and filter by quality. The same parameters for the specification of the adapter (5′- CGCCTTGGCCGTACAGCAG - 3′), minimum quality allowed (13 phred score), maximum allowed error rate (0.1), and colorspace mode were used for both the RNA-Seq and Ribo-Seq data. As for the length limitation, a size larger than 18 base pairs (bp) and a range between 25 and 40 bp were defined for RNA-Seq and Ribo-Seq data, respectively.

The Bowtie aligner (version 1.2.2) (Langmead et al., 2009) was used to remove contamination produced by readings of ribosomal RNA origin. *T. cruzi* rRNAs were downloaded from the TriTrypDB database. ShortStack (version 3.6) (Johnson et al., 2016) was used to align the previously obtained reads. The ShortStack program was adapted to accept data from SOLiD technology, while the prediction of secondary structures and micro RNAs was disabled. The mapping mode chosen was the single weighting mode (U) where, only the frequencies of the uniquely aligned reads in the vicinity of the alignment in question are considered in the final weighting. Then, the FeatureCounts module of the SubRead package (v1.5.2) (Liao et al., 2013) was used to quantify the number of reads originated in each transcript or CDS (for RNA-seq or Ribo-seq data, respectively). Finally, the translation efficiency in each stage was obtained with the RiboDiff software (Zhong et al., 2017).

### Generation of Sequence Logos

Sequence logos were generated with the WebLogo 3 online tool, using default parameters (Crooks et al., 2004).

### Gene Ontology Enrichment Analysis

Gene ontology analysis were performed using the tool available for this purpose in the TriTrypDB database for this purpose. The visualization and reduction of the categories was carried out by REVIGO (Supek et al., 2011) in conjunction with the graphic environment of the R language (R Core Team, 2013).

## Results and Discussion

### The *T. cruzi* Genome Contains Hundreds of uORFs With Repressive Potential

Precise definition of 5′UTR sequences was performed using the UTRme tool specifically developed to characterize untranslated regions of trypanosomatid genomes (Radio et al., 2018) using deep transcriptomic data obtained from Li et al. (2016). All open reading frames greater than three codons and present in the same strand as the CDS were then obtained. As described in the methods section, uORFs were classified as overlapping (uORFo) if the coding sequence starts in the 5′UTR region and ends within the main CDS (out of frame), or non-overlapping uORF (uORFno) which are contained entirely in the 5′UTR of the mRNA.

Since not all uORFs have coding potential nor do they have the same translation initiation efficiency, it is crucial to consider features that influence the repressive capacity of an uORF to define them. According to the literature, putative repressive uORFno elements are more likely to contain the following characteristics: (1) the presence of an AUG start codon (Clements et al., 1988); (2) a minimum distance of 15 nucleotides from the 5′ end, since shorter distances render difficult the assembly of the translation machinery at the uORF initiation codon (Vilela and McCarthy, 2003); (3) a maximum distance of 50 nucleotides between the stop codon of the uORF and the AUG of the main CDS, as shorter re-initiation times are associated to greater repressive potential (Chew et al., 2016) and (4) minimum length of 5 amino acids, as the longer the uORF length, the lower the probability of translation reinitiating at the main CDS (Kozak, 2001; Rajkowitsch et al., 2004). In the case of the uORFo, in which the coding sequences ends within the main CDS the requirements were: (1) to start with AUG; and (2) at least a minimum length of three amino acids before the AUG of the main CDS. Both types of uORFs passing the requirements were classified as repressive uORFs and 5′UTR regions that have one or more putative repressive elements were also classified as repressive. In turn, 5′UTRs that do not contain repressive elements, have a size greater than 50 nucleotides and do not contain AUGs were defined as non-repressive. Regions that do not fulfill any of the above categories were not assigned any classification.

After performing the above classification, the 5′UTR regions of *T. cruzi* mRNAs in both insect stages were studied. For the epimastigote stage, 6744 regions were analyzed, 568 of which (8.4%) classified as repressive. Among them, 111 regions contain only uORFo and 160 contain only uORFno, while the remaining regions (297) encompass elements of both categories. In addition, 3375 regions (50%) were classified as non-repressive. In the trypomastigote stage, 6750 mRNAs were analyzed, and a similar distribution was observed, 602 regions (8.9%) being classified as repressive. Among them, 231 carry both repressive types of uORFs, while 204 contain only uORFo and 167 only uORFno. Finally, 3333 regions (49%) were classified as non-repressive. These numbers are summarized in Table 1 and the list of gene identifiers for the analyzed UTRs can be found in Supplementary Table 1. Interestingly, the percentage of genes that have putative repressive elements is similar to that detected in *T. brucei* (11%) by Jensen et al. (2014). The observed differences with other reports (Vasquez et al., 2014; Fervers et al., 2018) are most likely due to the different inclusion criteria applied in each work.

**TABLE 1:** Number of genes presenting uORFs in *T. cruzi* epimastigotes and metacyclic trypomastigote stages.

***T. cruzi* life cycle stage**	**Repressive uORFs**			**Non-repressive**	**Total analyzed**
	**uORFo**	**uORFno**	**Both**		
Epimastigote	111	160	297	3375	6744
Trypomastigote	204	167	231	3333	6750

*The number of repressive (either overlapping, non-overlapping or both) and non-repressive uORFS are indicated. Also, the total number of transcripts analyzed for each *T. cruzi* stage is shown.*

### mRNAs Containing Putative Repressive uORFs Are Characterized by a Low TE in *T. cruzi*

**FIGURE 1:**
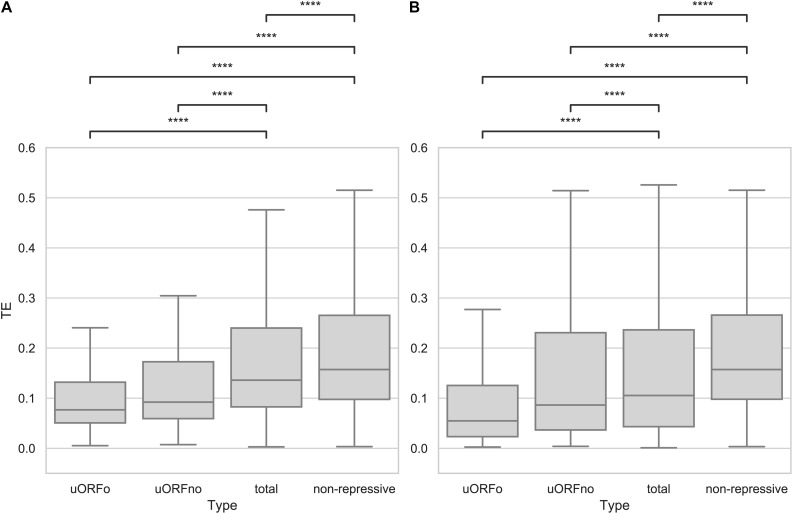
Translation efficiency of genes containing uORFs in *T. cruzi*. Box plots showing the distribution of TE values for genes with 5′UTR containing uORFo, uORFno, all genes and genes presenting UTRs classified as non-repressive. Statistically significant comparisons are indicated (Mann–Whitney *U p* < 1e-4 ****, *p* < 1e-3 ***, *p* < 1e-2 **, *p* < 0.05 *). **(A)** epimastigotes. **(B)** metacyclic trypomastigotes.

An open reading frame with repressive potential in the 5′UTR region of a gene could decrease its translation efficiency. To independently assess the repressive effect of the two defined categories, the TE values of the genes whose 5′UTR regions contain only repressive uORFno or only uORFo were calculated using Ribodiff and available ribosome profiling data obtained by our group (Smircich et al., 2015). TE values were also obtained for the non-repressive category and for all the genes. Translation efficiency values were then correlated to the presence of repressive uORFs in the 5′UTRs. The results show that the TE is indeed significantly correlated with the presence of uORFs that have repressive potential and with the subcategory to which they belong (Figure 1A).

Interestingly, genes containing only uORFo have the lowest TE. As ribosomes translating an uORFo are out of frame, they will read through the main CDS AUG, thus establishing an important translational control mechanism. The effect of naturally occurring uORFo has been poorly described in the literature, there are only a few previous reports on it (Wethmar, 2014). Recently, Fervers et al. observed that in *T. congolense*, as the distance between the uAUG and the AUG of the main CDS decreases, the translation efficiency does too, reaching the maximum decrease when the overlap occurs (Fervers et al., 2018), suggesting that this effect may be shared among trypanosomatids.

In the case of genes containing only uORFno, the drop in observed TE is lower than for those containing only uORFo. However, a clear repressive effect is seen, particularly when compared with the TE of the transcripts with 5′UTRs categorized as non-repressive. The TE of this later group is the highest in the entire comparison, suggesting that they indeed lack repressive uORFs and highlighting the relevance of these elements in translation control. In turn, the TE of all the 5′UTR regions shows an intermediate value between the repressive and non-repressive categories as expected considering the latter results.

In the metacyclic trypomastigote stage, a similar situation is evident even though the data show a higher dispersion, specially for the uORFno-containing genes (Figure 1B). It is worth noting that in the metacyclic trypomastigote stage, genome-wide translational repression has been described (Smircich et al., 2015), implying the existence of other regulatory mechanisms that exert a more significant effect on translation control.

It has been reported that the higher the number of uORFs in the 5′UTR of an mRNA, the higher the decrease in its translation efficiency (Chew et al., 2016). However, no correlation between the number of uORFs present in the 5′UTR region and the translation efficiency of the genes was found in our model (Supplementary Figure 1).

Globally, the results presented here are in good agreement with those previously obtained in other trypanosomatids, suggesting that the presence of open reading frames with regulatory potential in the 5′UTR regions is a contributing factor to translational control in these organisms. We also demonstrate that overlapping upstream open reading frames achieve the highest level of repression in *T. cruzi*. Additionally, the features we selected for uORF classification were confirmed to behave as good indicators of the repressive potential of uORFs, both in the case of repressive and non-repressive categories. Finally, we produced a dataset of *T. cruzi* genes where uORFs are likely acting as important regulatory elements.

### AUG Is the Main Initiation Codon of the Repressive uORF in *T. cruzi*

Pioneering studies initially proposed that uORFs with non-AUG initiation codons have poor translation efficiency (Clements et al., 1988). Later, several reports claimed that the majority of the translational initiation codons for uORFs were non-canonical (not AUG) (Ingolia et al., 2011; Fritsch et al., 2012; Lee et al., 2012). More recently, through the analysis of Ribo-seq data, it has been suggested that the translation efficiency of non-AUG initiating uORFs is low (Michel et al., 2014).

This led us to ask whether the presence of AUG as a start codon of an uORF can modulate the repressive effect on translation efficiency in *T. cruzi*. To find an answer to this question, we evaluated if uORFno initiating from each of the 61 codons would also cause a general effect on TE of the main CDS, as observed for AUG. Putative repressive uORFs were defined by maintaining the requirements to classify uORFs in this category (length, distance to the AUG, distance to the 5′ end) and only changing the criteria for the identity of the initiation codon. Thus, for each codon a set of uORFno was determined while preserving the rest of the requirements as before. Then, each new set of uORFs was correlated with the TE distribution of the genes, as done for before AUG initiating uORFs.

**FIGURE 2:**
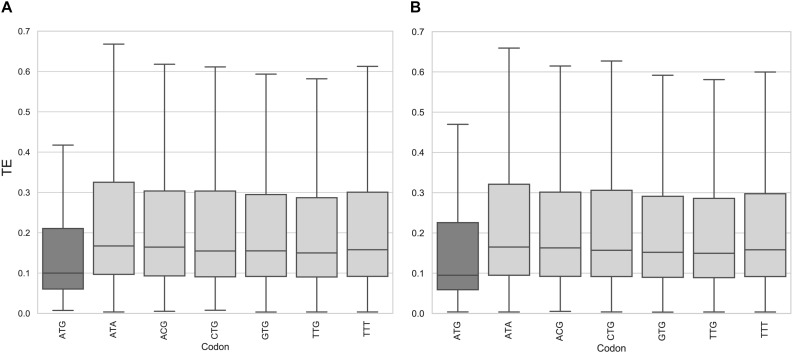
Translation efficiency of genes containing uORFno in *T. cruzi*. Box plots showing the distribution of translation efficiency values for genes with 5′UTR containing uORFno starting at each indicated codon. For each boxplot the procedure to identify uORFno with repressive potential was identical. TTT is used as a control codon (not described to efficiently initiate translation). **(A)** epimastigotes. **(B)** metacyclic trypomastigotes.

In the epimastigote stage, the translation efficiency of genes with uORFno using AUG as initiation codon is significantly lower (non-parametric Mann–Whitney *U* test < 0.01) compared to any other codon (Figure 2A and Supplementary Figure 2). Our results show no evidence of any particular behavior of mRNAs containing uORFs beginning with near cognate codons (NCC) known to be capable of translation initiation in other systems (CUG, UUG, GUG, ACG, AUA, and AUU) (Peabody, 1989; Ivanov et al., 2011). A similar situation is observed for the metacyclic trypomastigote stage (Figure 2B), the uORFno starting with AUG also being the only initiation codon associated with statistical significance to a low TE.

This approach allowed us to determine that AUG is the main initiation codon that generates repressive uORFno in *T. cruzi.* This finding further supports our original criteria. It is worth noting that this analysis does not eliminate the possibility that NCC initiate translation in specific uORFs with important regulatory consequences for the affected CDS. Indeed, experimentally assessing the translation of uORFs initiated by NCC would provide interesting insights in this regard. Even though Ribo-seq data has been used to this end in other models, the sensitivity reached by our data for the 5′UTR regions did not allow us to address this issue.

**FIGURE 3:**
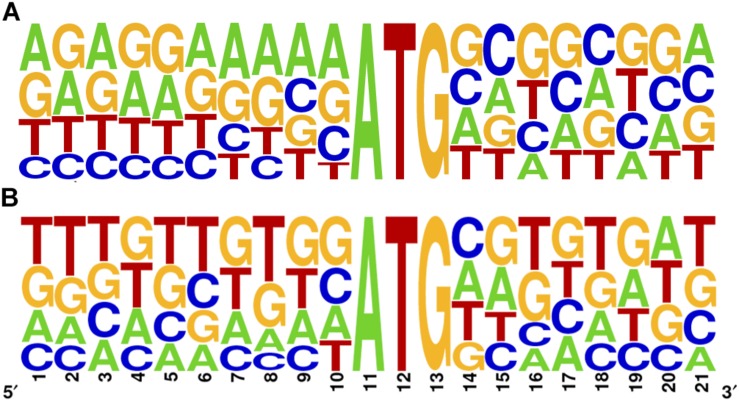
Sequence logo of the AUG initiator sequence in *T. cruzi*. 10 base pairs surrounding the A in position +1 are shown for the main CDS **(A)** and for the longest uORFno **(B)**. Context logo were generated with WebLogo (Crooks et al., 2004).

Kozak proposed that translation efficiency is strongly determined by the context of the initiating AUG (Kozak, 1978, 2002). In many organisms including *T. brucei* and *L. major*, only some of the characteristics defined by Kozak for mammalian cells are preserved, mainly the presence of A in the -3 position (Nakagawa et al., 2008). To further address this question in *T. cruzi*, we studied the primary sequence context of the initiator AUG of all main CDSs and repressive uORFno in the epimastigote stage (10 nt flanking the A in position +1 of the CDS). While the overrepresentation of A at position -3 is not as evident as for the other trypanosomatids, a clear purine enrichment is found for the main CDS AUG. However, this is not observed for the uORFs AUG (Figure 3), suggesting that initiation driven from the uAUG is not as efficient as from the main CDS AUG.

### The Presence of uORFno Is Correlated to the 5′UTR Length

**FIGURE 4:**
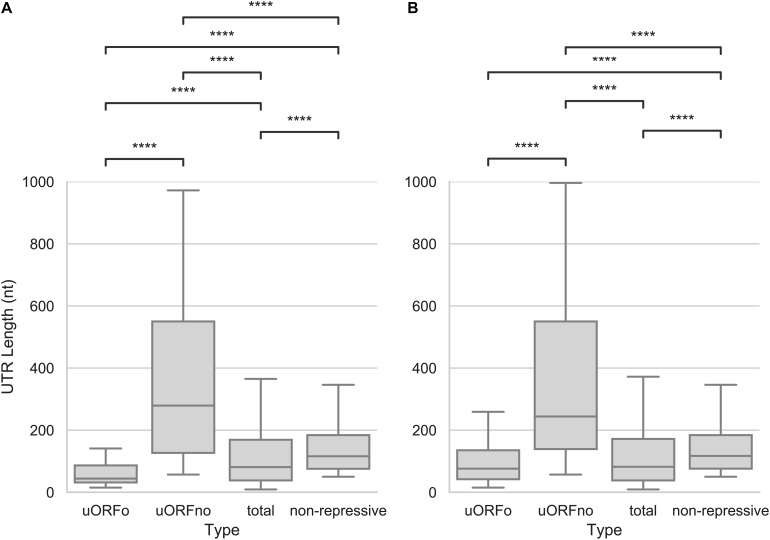
Length of the 5′UTRs in *T. cruzi*. Box plots showing the distribution of 5′UTR lengths for genes with 5′UTR containing uORFo, uORFno, all genes and genes presenting UTRs classified as non-repressive. Statistically significant comparisons are indicated (Mann–Whitney *U p* < 1e-4 ****, *p* < 1e-3 ***, *p* < 1e-2 **, *p* < 0.05 *). **(A)** epimastigotes. **(B)** metacyclic trypomastigotes.

As the presence of a uORFno will set a minimum length for the 5′UTR, we explored the size of this region. We found that the 5′UTR size of the uORFno associated genes is significantly larger (Mann–Whitney *U* test, *p*-value < 0.001) than the rest of the groups, for both the epimastigotes or metacyclic trypomastigote stages, as can be seen in Figure 4. Interestingly, the median size of repressive UTRs containing uORFno almost triples the length of the average *T. cruzi* 5′UTR. This finding implies that either the maintenance of uORFno increases the size of these regions, or that the length of the 5′UTR by itself is a determinant of the repressive potential. The latter is not a likely explanation as there is no general genome wide correlation between 5′UTR length and TE (Supplementary Figure 3).

In addition, this observation suggests that the minimum length of the 5′UTRs of genes regulated by uORFno might be evolutionary restricted to allow for the presence of the regulatory element. In support of this hypothesis, regions containing uORFo (elements that repress TE through a size independent mechanism), have the shortest 5′UTRs of all the groups.

### Genes Associated to Repressive 5′UTRs Have Low Expression in *T. cruzi*

**FIGURE 5:**
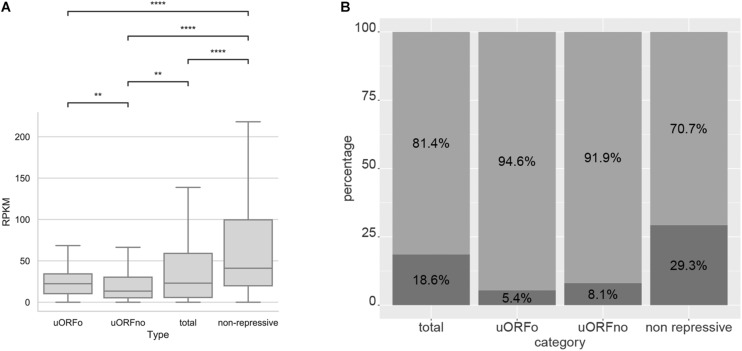
Analysis of the translation of genes with uORFs in *T. cruzi* epimastigotes. **(A)** Ribosomal footprints obtained from Smircich et al. (2015) were analyzed for genes with 5′UTR containing uORFo, uORFno, all genes and genes presenting UTRs classified as non-repressive. Boxplot of ribosomal footprints (RPKM) for genes in each category. Statistically significant comparisons are indicated (Mann–Whitney *U p* < 1e-4 ****, *p* < 1e-3 ***, *p* < 1e-2 **, *p* < 0.05 *). **(B)** Fraction of the genes belonging to each category present in the proteomic data of de Godoy et al. (2012) are represented in dark gray. A Fisher’s exact test was used to assess over or down representation of the number of detected proteins in proteomic experiments for each category compared to the number detected in the total proteome (all comparisons are significant *p* < 0.05).

In order to study the association between the presence of repressive uORFs and the translational level of the CDS, we comparatively analyzed the translation rates of genes containing repressive and non-repressive uORFs using our Ribo-Seq data. We found that genes with repressive uORFs have a decreased the number of ribosomal footprints compared to genes with non-repressive UTRs. Accordingly, the latter are also have the greatest translation rates (Figure 5A and Supplementary Figure 4A).

This effect is also reflected in the available proteomic data (de Godoy et al., 2012) where most proteins translated from genes containing repressive uORFs are not detected. Indeed, we found a significant underrepresentation of these genes in proteomic databases and an overrepresentation of proteins whose mRNAs do not contain repressive uORFs (Thermo Fisher Scientific test < 0.05, Figure 5B and Supplementary Figure 4B). As detection in proteomic studies is biased by the relative amount of protein in the sample, these data suggest low levels for proteins coded by repressive uORF containing mRNAs.

**FIGURE 6:**
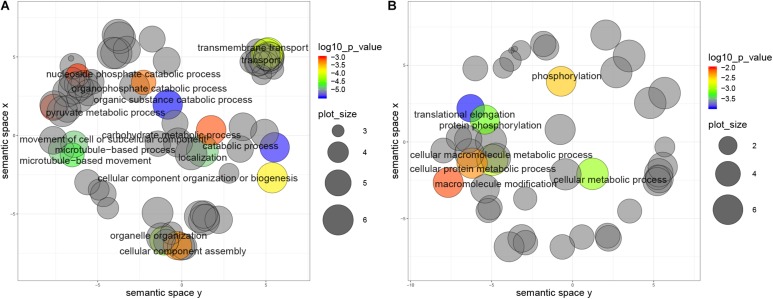
Biological process categories overrepresented in genes with uORFs in *T. cruzi* epimastigotes. Grouping reveals functionally related categories, size shows the frequency of the UniProtKB database category and color indicates a *p* value < 0.01. **(A)** Genes with non-repressive 5′UTRs, **(B)** Genes with repressive 5′UTRs.

Furthermore, genes with non-repressive 5′UTRs show ontology term enrichment with a trend for housekeeping functions such as catabolic processes, cell movement and transport, which are generally associated with high expression protein levels. Conversely, genes with 5′UTR containing repressive uORFs show enrichment in translational elongation and phosphorylation terms (Figures 6A,B). Similar results were obtained for the metacyclic trypomastigote stage, so we can conclude that this is not a stage specific characteristic (data not shown).

Finally, to evaluate if alternative 5′UTR processing could provide a mechanism to regulate the presence of uORFs (and thus the TE) between *T. cruzi* life stages, we searched for differential 5′UTR processing between epimatigotes and metacyclic trypomastigotes. First, we observed that most genes either share their main 5′UTR splice site (83% of the 3245 analyzed), or have sites are less than 10 nt apart (60% of the non-identical ones) (Supplementary Figure 5). This indicates that the presence or absence of uORFs is not a general mechanism to regulate differential TE between these life-cycle stages. We cannot discard that differential translation efficiency of the uORFs themselves might provide a regulatory mechanism of the main CDS. This requires further investigation as it cannot be assessed with our current data.

## Final Remarks

Overall, the results here presented allow us to conclude that uORFs are a frequent mechanism to fine tune translation efficiency in *T. cruzi*. Characteristics intrinsic to the uORF – such as its position within the 5′UTR, size and start codon – influence their ability to exert this effect. Genes with repressive uORFs have low levels of ribosomal footprints and are underrepresented in proteomic data, while the opposite is observed for genes with 5′UTR regions classified as non-repressive. Finally, differential 5′UTR processing does not seem to be a general mechanism to regulate their TE between the parasite life-cycle stages analyzed.

## Data Availability Statement

All datasets generated for this study are included in the article/Supplementary Material.

## Author Contributions

SR and PS designed the methodology. SR performed the analysis. JS-S, BG, and PS acquired the financial support. PS coordinated the project. All authors wrote and reviewed the manuscript.

## Conflict of Interest

The authors declare that the research was conducted in the absence of any commercial or financial relationships that could be construed as a potential conflict of interest.
