# Supplementary material for: Upstream ORFs Influence Translation Efficiency in the Parasite Trypanosoma cruzi
Source: Front Genet. 2020 Feb 28;11:166. doi: 10.3389/fgene.2020.00166 (PMC7059621; doi:10.3389/fgene.2020.00166)
Supplement: Supplementary file 1 [file Data_Sheet_1.docx]

Supplementary Material

# Supplementary Figures







A B

**Supplementary Figure 1. Correlation between the number of uORFs and the translation efficiency of the associated gene in *T. cruzi***

**A**. epimastigote stage **B**. metacyclic trypomastigote stage. A linear regression line is shown. The 95% confidence interval of the linear regression model is indicated in grey.





**Supplementary** **Figure 2. Translation efficiency of genes containing uORFno in *T. cruzi***

Box plots showing the distribution of translation efficiency values for genes with 5’UTR containing uORFno starting at each indicated codon. For each boxplot the procedure to identify uORFno with repressive potential was identical. Codon TTT (used as a random codon in figure 2) is indicated in dark grey. **A.** epimastigotes. **B.** metacyclic trypomastigotes.


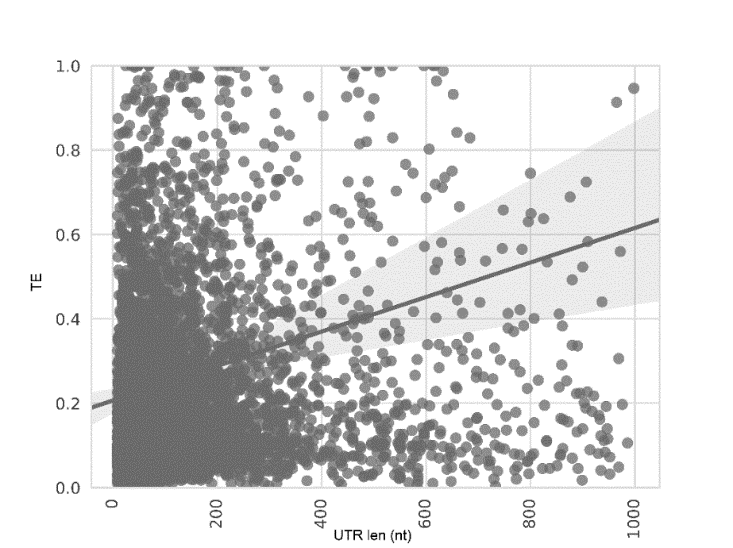

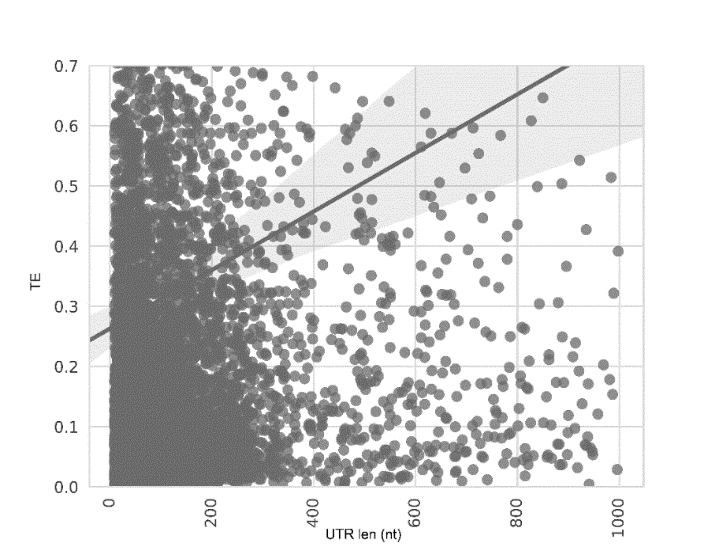


A B

**Supplementary Figure 3**. **Correlation between 5’UTR size and translation efficiency of *T. cruzi* genes**

**A**. epimastigote stage, **B**. metacyclic trypomastigote stage. A linear regression line is shown. The 95% confidence interval of the linear regression model is indicated in grey.


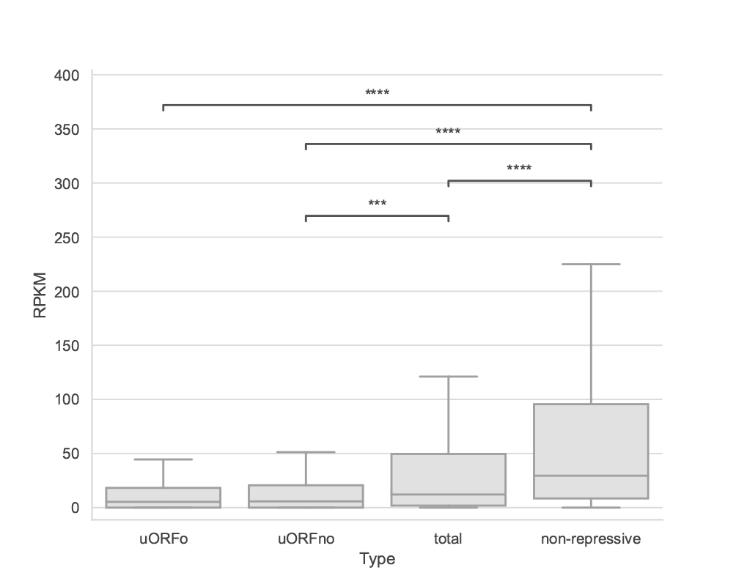

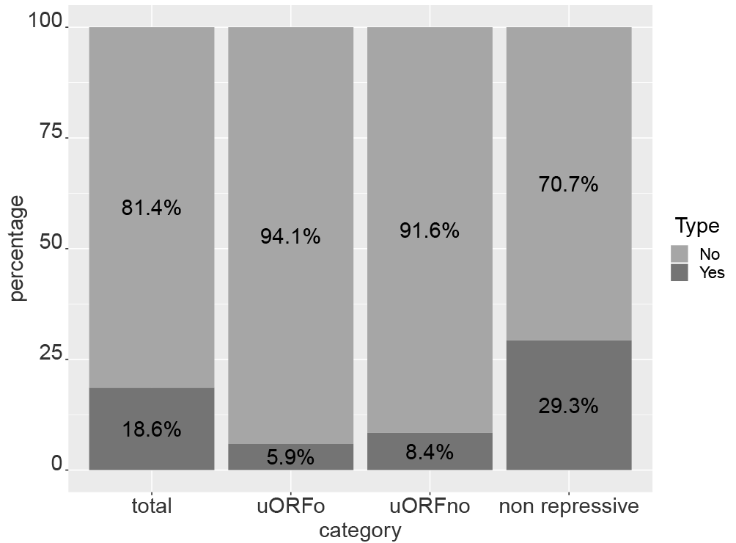


A B

**Supplementary Figure 4. Analysis of the translation of genes with uORFs in *T. cruzi* metacyclic trypomastigotes**

Ribosomal footprints obtained from (Smircich et al., 2015) were analyzed for genes with 5’UTR containing uORFo, uORFno, all genes and genes presenting UTRs classified as non-repressive **A**. Boxplot of ribosomal footprints (RPKM) for genes in each category, **B**. fraction of the genes belonging to each category present in the proteomic data of (de Godoy et al., 2012). A Fisher's exact test was used to assess over or down representation of the number of detected proteins in proteomic experiments for each category compared to the number detected in the total proteome (all comparisons are significant p < 0.05).


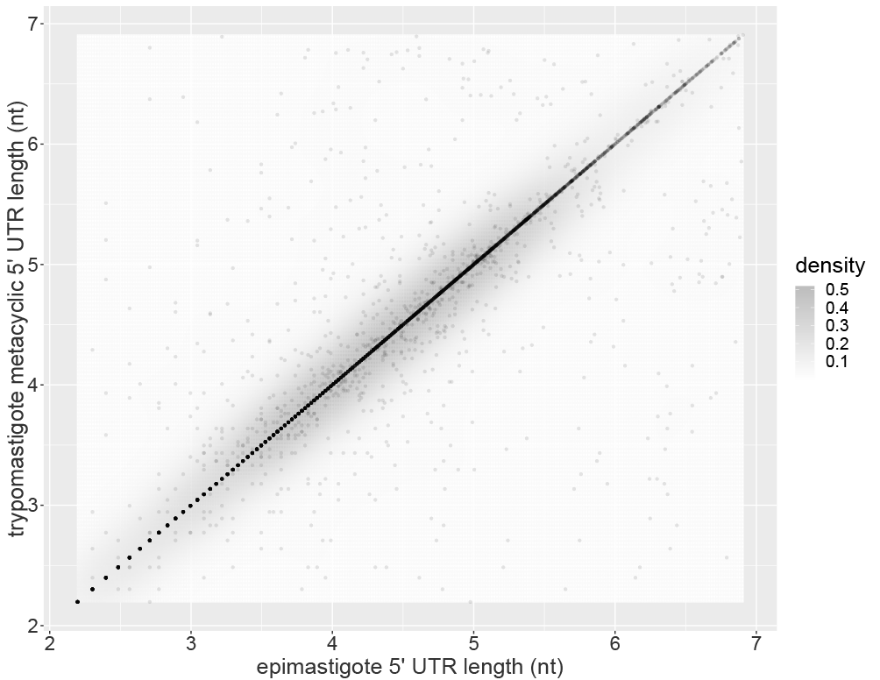


**Supplementary Figure 5. Comparison of the 5'UTR size between epimastigotes and metacyclic trypomastigotes**
